# Structural Characterization and Ligand-Induced Conformational Changes of SenB, a Se-Glycosyltransferase Involved in Selenoneine Biosynthesis

**DOI:** 10.1021/acs.biochem.3c00452

**Published:** 2023-11-15

**Authors:** Kendra A. Ireland, Chase M. Kayrouz, Jonathan Huang, Mohammad R. Seyedsayamdost, Katherine M. Davis

**Affiliations:** † Department of Chemistry, Emory University, Atlanta, Georgia 30322, United States; ‡ Department of Chemistry, Princeton University, Princeton, New Jersey 08544, United States; § Department of Molecular Biology, Princeton University, Princeton, New Jersey 08544, United States

## Abstract

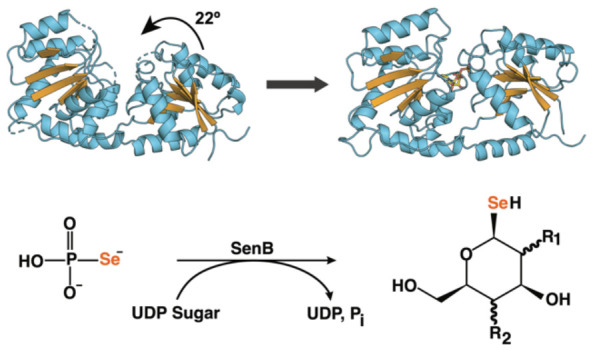

Selenium (Se) is an essential micronutrient that is found naturally in proteins, nucleic acids, and natural products. Unlike selenoproteins and selenonucleic acids, little is known about the structures of biosynthetic enzymes that incorporate Se into small molecules. Here, we report the X-ray crystal structure of SenB, the first known Se-glycosyltransferase that was recently found to be involved in the biosynthesis of the Se-containing metabolite selenoneine. SenB catalyzes C–Se bond formation using selenophosphate and an activated uridine diphosphate sugar as a Se and glycosyl donor, respectively, making it the first known selenosugar synthase and one of only four *bona fide* C–Se bond-forming enzymes discovered to date. Our crystal structure, determined to 2.25 Å resolution, reveals that SenB is a type B glycosyltransferase, displaying the prototypical fold with two globular Rossmann-like domains and a catalytic interdomain cleft. By employing complementary structural biology techniques, we find that SenB undergoes both local and global substrate-induced conformational changes, demonstrating a significant increase in α-helicity and a transition to a more compact conformation. Our results provide the first structure of SenB and set the stage for further biochemical characterization in the future.

Selenium is a trace element and essential micronutrient that occurs naturally in proteins and nucleic acids in the form of selenocysteine and selenouridine, respectively.^1^ Its antioxidant and anti-inflammatory properties play a multitude of protective roles in human health, especially for the prevention of conditions associated with oxidative stress.^2^ While the biosynthetic pathways for selenoproteins and selenonucleic acids are well-known,^3,4^ the incorporation of Se into small molecules is poorly understood. Kayrouz et al. recently characterized a widespread three-gene cluster responsible for the biosynthesis of selenoneine,^5^ the Se analogue of ergothioneine, a naturally occurring sulfur (S)-containing amino acid that functions as a potent antioxidant and cytoprotectant.^6^ Many biosynthetic enzymes are unable to discriminate between S and Se due to their similar valency, electronegativity, and size. In fact, S-utilizing enzymes often display slightly lower Michaelis constants (*K*_m_) for seleno-isologues, yielding nonspecific selenometabolites.^7,8^ Prior to the work by Kayrouz and co-workers, selenoneine’s natural origin was assumed to result from nonspecific incorporation by the ergothioneine pathway. However, the identification of the selenoneine gene cluster (*sen*) revealed the first dedicated pathway for the production of a selenometabolite.

Examination of the *sen* cluster led to the characterization of three enzymes, SenC, SenB, and SenA. By converting intracellular selenide to selenophosphate (SeP), SenC generates the Se donor in this pathway (Figure 1A). SenB then uses SeP and activated nucleotide sugars as glycosyl donors to form selenosugars through the generation of a C–Se bond. Finally, SenA introduces a second C–Se bond between the newly generated selenosugar and l-hercynine. Spontaneous *syn* elimination of the resulting selenoxide releases the sugar moiety, and a final reduction step furnishes selenoneine (Figure 1A). Of the enzymes in this pathway, SenB is especially noteworthy, as its unusual ability to form selenoglycosidic linkages expands the scope of glycosylation reactions in nature and further underlines the notion that it belongs to a new enzyme family annotated as TIGR04348 by the NCBI, for which structures are not yet available.

**Figure 1:**
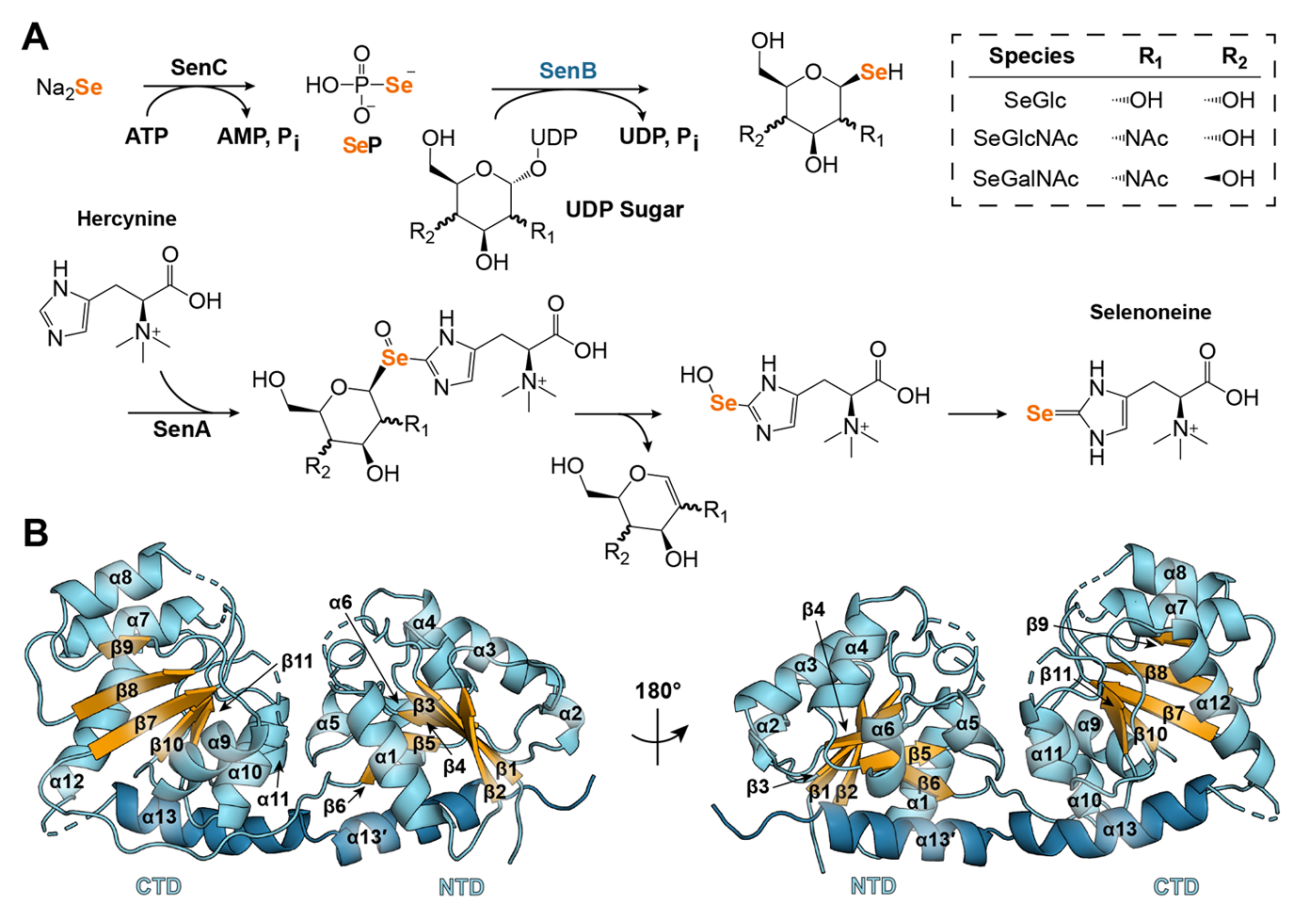
Selenosugar synthase SenB is involved in the biosynthesis of the antioxidant selenoneine. (A) Biosynthetic pathway of selenoneine. (B) Overall architecture of SenB. Domains and secondary structure elements are labeled. The C-terminal α-helix that traverses the molecule is colored dark blue. All other β-strands and α-helices/loops are shown in orange and light blue, respectively.

To gain insights into the structural basis for SenB’s unique reactivity, we generated crystals of the *Variovorax paradoxus* orthologue and determined its structure to 2.25 Å resolution [Protein Data Bank (PDB) entry 8FBX (see the Supporting Information for details and Table S1 for data processing and refinement statistics)]. Despite a lack of significant sequence homology to any structurally characterized proteins, the overall SenB architecture displays the canonical glycosyltransferase type B (GT-B) fold, consisting of two globular Rossmann-fold domains (Figure 1B).^9,10^ Each domain comprises a parallel β-sheet linked to six short α-helices. While the β-sheet of the N-terminal domain (NTD) is comparatively extended to six strands in length, the C-terminal domain (CTD) terminates in a long α-helix, which extends across the entire molecule. The orientation of the two domains forms an electropositive interdomain cleft (Figure S1A) known to house the active site in other nucleotide sugar-binding glycosyltransferases. Further inspection reveals a second electropositive pocket, ∼20 Å away, that is linked to the putative active site by a positively charged groove (Figure S1B). It is possible that this Arg-rich site may also play a role in catalysis, perhaps as an exosite for the nucleotide sugar. Unlike the glycosyltransferase type A (GT-A) fold, GT-B enzymes are typically metal-independent and lack the D/EXD metal-binding motif present in GT-As.^11^ Activity assays confirmed that SenB does not require a divalent metal for activity (Figure S2), and no density was observed for a bound ion.

Following crystallographic characterization, we used the Dali server to determine SenB’s closest structural homologues from proteins deposited in the PDB (Table S2).^12^ Unsurprisingly, a series of glycosyltransferases ranked highly in this analysis. These enzymes are known to display pronounced structural similarity despite significant sequence diversity, and SenB is no exception.^11^ Its structure closely resembles the sucrose phosphate synthase (SPS) from *Thermosynechococcus elongatus* (PDB entry 6KIH)^13^ with an overall Cα root-mean-square deviation (RMSD) of 2.6 Å despite a level of sequence identity of only 18%. More in-depth sequence comparison of these structural homologues reveals that SenB’s CTD residues are more highly conserved than its NTD residues (Figure S3), consistent with the general substrate-binding motifs of this enzyme class, namely, that widely variable acceptor substrates interact primarily with the NTD,^9,14^ while activated nucleotide sugars predominantly bind to residues within the CTD.^15,16^

SenC was previously shown to discriminate against S incorporation as it does not accept sodium sulfide as a substrate. We similarly probed the Se specificity of SenB and found a 10–20-fold discrimination against thiophosphate (Figure S4). The enzyme also shows a preference for uridine diphosphate *N*-acetylglucosamine (UDP-GlcNAc), when compared to UDP-glucose or UDP-*N*-acetylgalactosamine.^5^ To gain structural insights into substrate binding, we soaked SenB crystals with UDP or UDP-GlcNAc; however, this treatment dramatically reduced the crystal quality, in many cases causing rapid dissolution that precluded structural characterization. Co-crystallization was equally unsuccessful. As interdomain flexibility is an essential characteristic of nucleotide sugar recognition by many glycosyltransferases, we hypothesize that this behavior suggests the potential for UDP/UDP-GlcNAc-induced conformational rearrangements that are not supported by the current crystal lattice.^11,17^ GT-B enzymes, in particular, commonly undergo reorientation of the two globular domains upon nucleotide sugar binding.^18−20^ The most dramatic example of this is MshA (PDB entries 3C48, 3C4Q, and 3C4V), whose NTD rotates ∼97° upon UDP binding to generate the binding site for the acceptor substrate (Figure 2A).^21^ The apparent disorder of SenB’s interdomain cleft in the absence of a substrate (Figure 1B, regions of missing electron density indicated by dashed lines) is analogous to that observed for substrate-free MshA, perhaps consistent with catalytically relevant conformational flexibility. By contrast, efforts to obtain a SeP or thiophosphate-bound crystal structure of SenB simply yielded structures lacking ligand density, suggesting that in the absence of a nucleotide sugar, the phosphate group may not bind to the enzyme’s open conformation.

**Figure 2:**
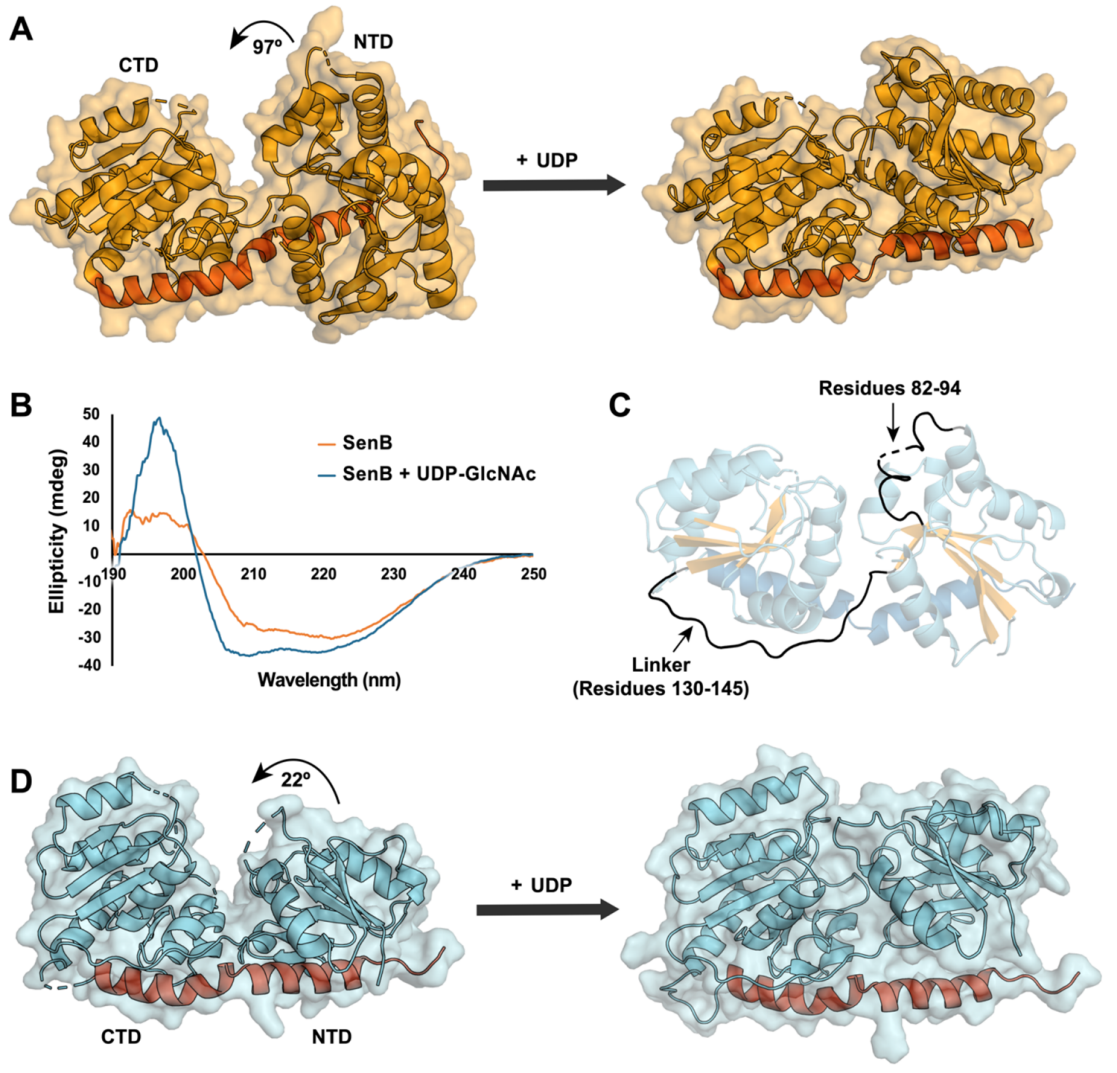
UDP/UDP-GlcNAc-induced structural changes in SenB and MshA. (A) Binding of UDP to MshA (PDB entries 3C48 and 3C4Q) triggers a 97° rotational reorientation of the NTD. (B) CD spectra demonstrate a significant increase in the level of α-helical character upon the addition of UDP-GlcNAc to SenB. (C) Flexible loops in SenB proposed to become partially stabilized upon UDP-GlcNAc binding. (D) *In silico* docking experiments indicate that binding of UDP to SenB induces a 22° shift of the NTD to a closed conformation.

To further investigate the conformational dynamics involved in nucleotide sugar recognition, we employed circular dichroism (CD) spectroscopy and small-angle X-ray scattering (SAXS). Unlike X-ray crystallography, these methods provide information about biologically relevant conformations within a native aqueous environment. CD spectra demonstrate a change in tertiary structure upon UDP-GlcNAc addition, with an estimated 7.6% increase in the level of α-helical character predicted by the BeStSel Web server (Figure 2B).^22^ We hypothesize that the observed increase in α-helicity is due to ordering of otherwise unstructured loops, which link the two domains (residues 130–145) and lie at the domain interface [NTD residues 82–94 (Figure 2C)], to form a more compact form of the enzyme upon nucleotide sugar binding.

Guinier plots (Figure S5A) and pair distance distribution functions (Figure S5B) generated from SAXS measurements reveal a corresponding ∼1.1 Å decrease in the radius of gyration (*R*_g_) and a 10 Å decrease in the maximum diameter (*D*_max_) upon UDP-GlcNAc binding (Table S3). Although a slight reduction in size is visible, the shape of the molecular envelope does not change significantly with the addition of UDP-GlcNAc (Figure S5C). It seems likely that while UDP/UDP-GlcNAc appear to induce conformational changes in SenB (Figure 2D), they are not nearly as significant as those observed in MshA, which undergoes a 2.4 Å reduction in *R*_g_ upon substrate binding (as calculated by HullRad).^21,23^

With UDP-GlcNAc-triggered domain movement in mind, we proceeded to investigate the mode of ligand binding through molecular docking simulations (see the Supporting Information for details). We first simulated a catalytically active conformation by separately docking the two domains, which yielded a structure demonstrating an ∼22° rotational reorientation of the NTD (Figure 2D). UDP and SeP were sequentially docked to the resulting closed SenB structure. GT-B enzymes often have a key C-terminal Glu residue that interacts with the ribose moiety of the nucleotide donor and Gly-rich loops that interact with the phosphate moieties.^9^ In SenB, this ribose-binding residue appears to be E239, which forms a hydrogen (H)-bond to a hydroxyl group of the ribose ring in the docking model (Figure 3A). R22 forms additional H-bonds to the ribose hydroxy groups, while N17, L209, and T214 coordinate to the uracil ring. G19, N20, R155, and E231 appear to be involved in binding to the phosphate moieties via several H-bonds. Residues N20, R155, E231, and E239 are all highly conserved among SenBs from various organisms, lending credence to their involvement as key UDP-binding contacts. The SenB docking model resembles the UDP-bound structures of MshA (Figure 3B), SPS (Figure S6B), and BshA from *Bacillus anthracis*(24) (Figure S6C), another structural homologue of SenB (see Table S2). In each example, UDP binds in a similar orientation at the interface of the two domains (Figure S7). More specifically, G19 and R155 in SenB appear to be analogous to the phosphate-binding Gly and Arg/Lys residues in the other GT-Bs, while H-bonds between the backbone of L209 in SenB and the uridine ring are similar to the backbone-mediated H-bonds for R294 in MshA, I309 in SPS, and Q262 in BshA.

**Figure 3:**
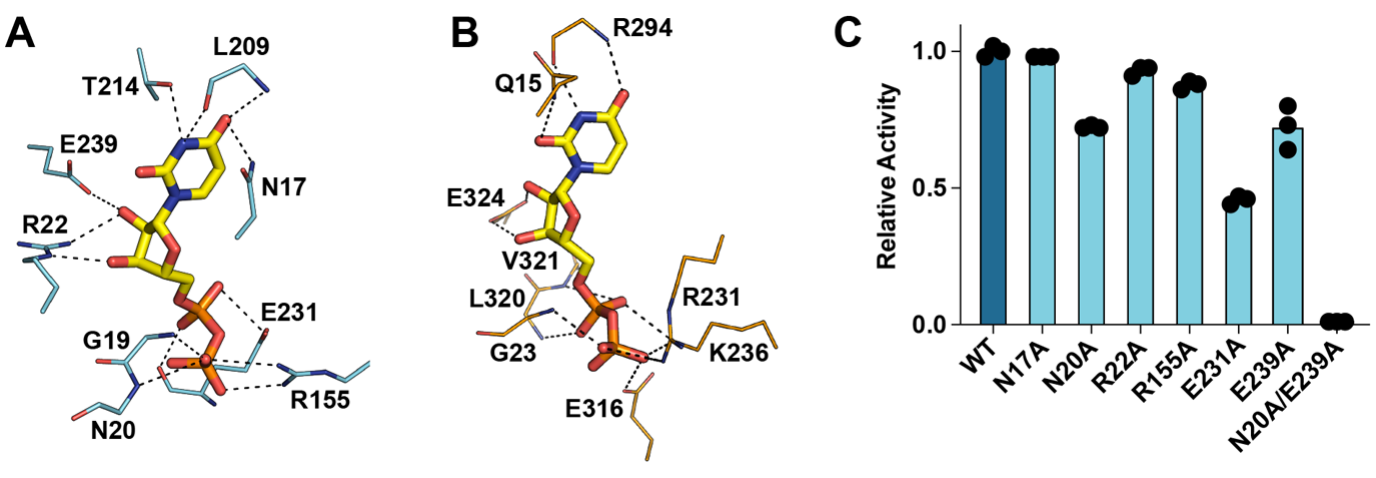
Predicted UDP binding mode in SenB compared to MshA. Shown are (A) the SenB docking model and (B) UDP-bound MshA (PDB entry 3C4V). (C) Catalytic activity of SenB variants relative to that of the wild type. The averages of three independent measurements are shown.

Our docking model suggests the involvement of additional UDP-binding residues in the NTD relative to MshA, SPS, and BshA. Comparison of the docking model and crystal structure demonstrates that substrate-binding contacts from the NTD are possible only in the closed conformation due to rotation of α1 (containing all NTD residues implicated in UDP binding) and the loops surrounding α3 and β4 (responsible for binding SeP) (Figure S8). This analysis suggests that SenB’s specific conformational dynamics may be responsible for the NTD’s more significant role in nucleotide sugar binding compared to other glycosyltransferases. Mutation of residues N20, R22, R155, E231, and E239 to Ala demonstrated a moderate reduction in enzymatic activity in support of our model (Figure 3C). Further examination reveals a continuation of the electropositive surface adjacent to the docked UDP. This hydrophilic pocket aligns with similar features of MshA known to bind its phosphate-containing cosubstrate.^21^ Our simulations predict this pocket as the site of SeP binding in SenB via H-bonding interactions with residues N20, H58, R61, T83, T85, and R155 (Figure 4). To validate this binding mode, we generated an N20A/E239A variant that displayed an almost complete loss of activity (Figure 3C).

**Figure 4:**
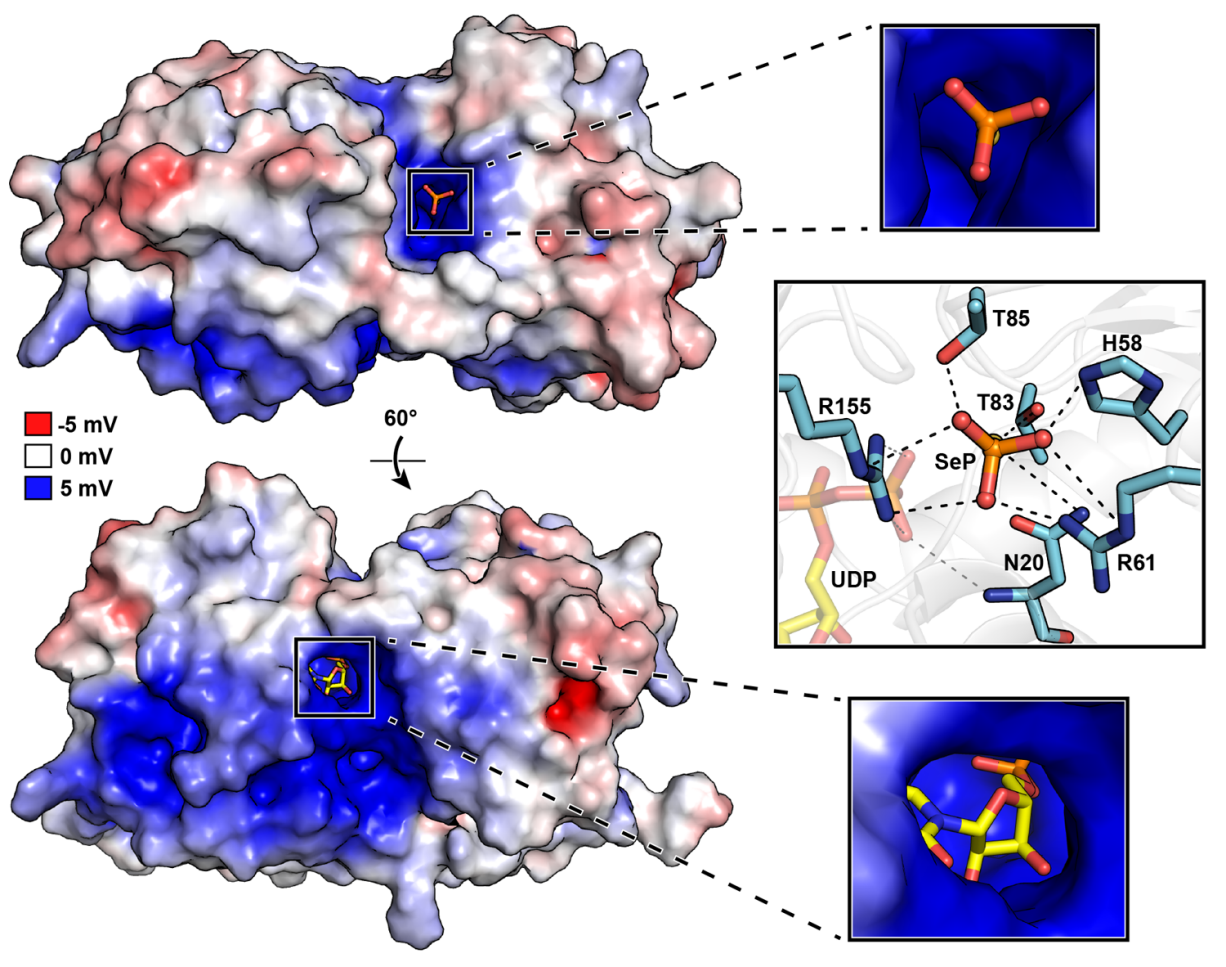
Electrostatic surface representation of the docking model and predicted binding mode of SeP.

In conclusion, we describe herein the structure of the first characterized selenosugar synthase involved in the biosynthesis of selenoneine. Despite a pronounced lack of sequence similarity to characterized glycosyltransferases, SenB displays a characteristic GT-B fold. Complementary techniques point to changes in the tertiary structure of SenB upon UDP-GlcNAc binding. On the basis of these results, we propose that UDP-GlcNAc first binds to the CTD of SenB, prompting a shift to a more globular, closed conformation via rotation of the NTD and stabilization of active site loops into ordered α-helices. We hypothesize that closing of the large interdomain cleft in response to glycosyl donor binding subsequently generates the binding site for SeP by bringing the acceptor substrate-binding NTD residues closer to the nucleotide sugar. Altogether, our results shed light on SenB’s structure in the open conformation, reveal the presence of ligand-induced conformational changes, and identify potential ligand-binding contacts through docking and mutagenesis experiments. This work provides a starting point for elucidating the remaining mysteries surrounding SenB and other yet-to-be-discovered Se-glycosyltransferases.

## Supporting Information Available

Detailed description of materials and methods, crystallographic and refinement statistics, Tables S1–S3, and Figures S1–S8 (PDF)

## Accession Codes

PDB entry 8FBX and NCBI entry WP_080642484.1.
