# Supplementary material for: Structural Characterization and Ligand-Induced Conformational Changes of SenB, a Se-Glycosyltransferase Involved in Selenoneine Biosynthesis
Source: Biochemistry. 2023 Nov 15;62(23):3337–42. doi: 10.1021/acs.biochem.3c00452 (PMC10702425; doi:10.1021/acs.biochem.3c00452)
Supplement: Supplementary file 1 — bi3c00452_si_001.pdf [file bi3c00452_si_001.pdf]

Supplementary Information for

Structural Characterization and Ligand-Induced Conformational Changes of SenB, a Se-Glycosyltransferase Involved in Selenoneine Biosynthesis

Kendra A. Ireland,[1] Chase M. Kayrouz,[2] Jonathan Huang,[2] Mohammad R. Seyedsayamdost,[2,3] and Katherine M. Davis[1,*]

[1]Department of Chemistry, Emory University, Atlanta, GA 30322, United States

[2]Department of Chemistry, Princeton University, Princeton, NJ 08544, United States

[3]Department of Molecular Biology, Princeton University, Princeton, NJ 08544, United States

*Correspondence: katherine.davis@emory.edu

Materials and Methods

Materials

Escherichia coli strains DH5α and BL21(DE3) were purchased from New England Biolabs (Ipswich, MA). Unless stated otherwise, all materials were purchased from Sigma Aldrich (St. Louis, MO).

6xHis-SenB Protein Sequence

MGSSHHHHHHSSGLVPRGSHMSNPSLVIVSPALPGANNGNWRTAQRWKALLSPVCSARVVQQWPDADA
SADTVMLALHARRSAESIAHWAHAHPGRGLGVVLTGTDLYQDIGSDPQAQRSLQLAQRLVVLQALGAEALP
PECRAKARVVYQSTSARAELPKSARQLRAVMVGHLRQVKSPQTLFDAARLLCGREDIRIDHIGDAGDAGLGE
LARALASDCPGYRWLGALPHAQTRQRIQRAHVLVHTSALEGGAHVIMEAVRSGTPVLASRVPGNVGMLGN
DYAGYFPHGDAAALAALLEACRAGQGSKDRAAGLLDSLRTQCALRAPLFDPRAEQAALFQLLNELQPPPP

Protein Expression and Purification

Construction of pET28b(+) vectors, expression, and Ni-NTA purification of 6xHis-tagged SenB (NCBI accession number WP_080642484.1) and SenC (NCBI accession number WP_062361881.1) proteins from *Variovorax paradoxus* DSM 30034 were carried out as previously described.[1] SenB variants were co-expressed with the GroEL-ES chaperone encoding plasmid pGro7 (Takara Bio) and purified in otherwise identical fashion. For crystallography, wild-type SenB was further purified by fractionation on a Sephacryl S-200 HR HiPrep 16/60 column (Cytiva) with a running buffer consisting of 20 mM Tris, 100 mM NaCl, 10% glycerol, pH 8. Purified proteins were stored at −80 °C. Protein concentrations were determined spectrophotometrically on a Cary 60 UV-visible spectrophotometer (Agilent) using an ExPASy-calculated molar extinction coefficient at 280 nm.

Chemical Synthesis of Selenophosphate

The literature procedure reported by Chojnowski and Cypryk was followed.[2] A 50 mL round-bottom flask equipped with a Teflon stir bar, a water condenser, and a rubber septum was charged with tris(trimethylsilyl)phosphite (5.97 g, 20 mmol) and selenium powder (1.66 g, 21 mmol). The resulting mixture was heated to 50 ^{o}C in a pre-heated oil bath for 1 h. After cooling, the remaining selenium was removed by filtration to afford *O*,*O*,*O*-tris(trimethylsilyl) phosphoroselenoate (7.08 g, 94%) as a colorless liquid, which was used for the next step without further purification.

The literature procedure reported by Glass and Skowronska was followed.[3] A 50 mL round-bottom flask equipped with a Teflon stir bar and a rubber septum was charged with *O*,*O*,*O*-tris(trimethylsilyl) phosphoroselenoate (377.5 mg, 1.0 mmol) and hexanes (10 mL). The resulting solution was degassed by bubbling with N_2 for 10 min, at which point N_2 degassed tricine buffer (10 mL) was added. The resulting biphasic mixture was stirred vigorously at room temperature for 12 h. Layers were then separated. The aqueous layer was transferred to a glass vial under N_2 and used directly for the enzymatic assay.

Enzyme Activity Assays

All enzyme assays were carried out in an MBraun glovebox under a N_2 atmosphere maintained at <0.1 ppm O_2. HPLC–MS was performed on an Agilent instrument equipped with a 1260 Infinity Series HPLC, an automated liquid sampler, a photodiode array detector, a JetStream ESI source, and the 6540 Series Q-tof mass spectrometer. HPLC–MS data were acquired and analyzed with Agilent MassHunter software. Solvents for all HPLC–MS experiments were water and 0.1% formic acid (solvent A), and MeCN and 0.1% formic acid (Solvent B). General assay procedures were as previously described[1] (see SI figure legends for more details). Reactions were quenched and products were derivatized by the addition of an equal volume of 10 mM monobromobimane (mBBr) in MeCN followed by a 30 min incubation period at room temperature to allow for complete derivatization. Reactions were analyzed by HPLC–MS using a Synergi Hydro-RP column (Phenomenex, 250 × 4.6 mm, 4 µm) with a flow rate of 1 mL min^{-1} and an elution program consisting of a 5% solvent B wash for 3 min, a gradient of 5–75% solvent B over 6 min, followed by a gradient of 75–100% solvent B over 1 min, and a final hold at 100% for 5 min.

For the activity assays in Fig. 3C, reactions were carried out in 50 mM Tris buffer (pH 8) containing 2 mM DTT, 1 mM UDP-GlcNAc, 1 mM synthetic SeP, and 20 µM wild-type or mutant SenB. Reactions were performed in triplicate. After 60 min, reactions were derivatized with mBBr and analyzed by HPLC, monitoring absorbance at 390 nm. Relative activity is the ratio of product peak area of a SenB variant to that of wild-type.

Protein Crystallization

Crystals of SenB were grown using the sitting drop vapor diffusion method at room temperature. 19 mg mL^{-1} SenB with 5 molar eq. of $MnCl_2$ in 20 mM Tris, 100 mM NaCl, 10% glycerol, pH 8 was sonicated in an ultrasonic ice water bath for 10 min to remove O_2, then left in an anaerobic chamber for an additional 4 h to ensure anaerobicity. 10 eq. of *N*-acetyl-1-seleno-β-D-glucosamine (SeGlcNAc – synthesized as previously described)[1] and 10 mM sodium dithionite were then added to the protein solution. To obtain crystals, the protein solution was mixed 1:1 with a precipitant solution of 0.1 M Bis-Tris propane pH 7, 2.7 M sodium formate. Crystals displaying a many-sided polyhedron morphology appeared within 2-4 weeks and were fully formed within 2 months. A streak seeded tray was then set up for the same condition using 30 eq. of SeGlcNAc, and the crystals harvested from the seed tray for data collection. For crystal harvesting, the crystals were looped and briefly transferred into cryoprotectant, comprised of the precipitant with 28.8% (v/v) ethylene glycol, before flash freezing in liquid N_2.

X-ray Data Collection and Processing

$MnCl_2$ was added to the protein solution prior to crystallization to allow for experimental phasing using the anomalous signal at the Mn^{2+} K-edge, as using the NCBI Basic Local Alignment Search Tool (BLAST)[4] for the amino acid sequence of SenB against the entire PDB yielded no results that could be used as molecular replacement models. Many glycosyltransferases require a divalent cation for catalysis (usually Mg^{2+} or Mn^{2+}), and without knowledge of SenB's specific fold, we suspected that it may also bind a divalent cation. In case Mn^{2+} did not bind to SenB and/or in the absence of sufficient anomalous signal for structure solution, SenB's enzymatic product, SeGlcNAc, was included to allow for phasing with the Se K-edge. Data was therefore collected above both the Mn^{2+} edge (6.6 keV, 1.8785 Å) and the Se edge (12.67 keV, 0.9785

Å). Unfortunately, an anomalous correlation was not observed in any of the diffraction data, and no density was observed for SeGlcNAc or a bound ion in the final structure, leading us to believe that SenB is a metal-independent enzyme. The determination that SenB possesses a GT-B glycosyltransferase fold supports this assignment, as such enzymes (unlike those with a GT-A fold) generally do not require bound metal ions for catalysis.[5, 6] Subsequent activity assays (Fig. S2) confirmed that SenB does not require a divalent metal for activity.

Diffraction data were collected with an incident wavelength of 0.9785 Å at beamline 23-ID-B (GM/CA) of the Advanced Photon Source at Argonne National Laboratory using an Eiger X 16M (Dectris) detector. Crystals were maintained at 100 K to minimize X-ray-induced damage while images were collected sequentially ($\Delta\phi$ = 0.2°). The data were subsequently indexed, integrated, and scaled using XDS before merging with *AIMLESS*.[7, 8] In the absence of an anomalous signal, the structure of SenB was solved via molecular replacement with *PHASER* using the AlphaFold2 (AF) predicted structure as the search model,[9, 10] after removing all residues from the model with per-residue confidence scores (pLDDT) below 90%.[11] The resulting modified AF model consisted of residues 25-30, 43-103, 106-161, 164-204, 207-250, 254-305, and 315-346, or ~83% of SenB's total amino acids. Note that AF confidently predicted the conserved core structure of the GT-B fold, while the active site cleft involved in ligand-binding and ligand-induced conformational rearrangements was significantly more ambiguous, and many associated residues had to be removed for phasing. More specifically, many low-confidence residues were located within the first α-helix, predicted to rotate inward toward the CTD upon UDP binding (see Fig. S8). This structural feature houses all of the NTD residues that our docking model predicts are involved in UDP binding (i.e. N17, G19, N20, and R22). Another residue implicated in nucleotide sugar binding, E231 from the CTD, is also located in a low-confidence region.

Crystallographic model building was conducted in Coot,[12] the structure refined in Phenix,[13] and the model quality assessed using Molprobity.[14] The SenB structure is in the P 1 2 1 space group and contains three molecules in the asymmetric unit. Analysis of crystallographic symmetry reveals that lattice contacts occur distal to the interdomain cleft and do not appear to influence the overall conformation of the enzyme. Interactions between the C-terminal α-helices of symmetry mates dominate, with the majority of contact occurring in the NTD. The final model (PDB ID: 8FBX) was refined to 2.25 Å resolution. Selected data processing and refinement statistics can be found in Table S1. Figures depicting the structure were generated with PyMOL.[15] The electrostatic surface representations shown in Fig. 4 and S1 were generated using the Adaptive Poisson-Boltzmann Solver (APBS) electrostatics plugin in PyMOL.[16]

Circular Dichroism Spectroscopy

CD spectra were recorded on a Jasco J-1500 CD spectropolarimeter using a 0.1-mm path length quartz cuvette. Spectra were collected on 100 µM SenB containing 1 molar eq. of $MgCl_2$ in 20 mM Tris, 100 mM NaCl, 10% glycerol, pH 8. 5 molar eq. of UDP-GlcNAc was included for the substrate-bound sample. Three spectra were collected and averaged over a wavelength range from 190 to 250 nm at a scanning rate of 100 nm min^{-1} and a bandwidth of 1 nm. All spectra were corrected for the solvent contribution.

Small-Angle X-ray Scattering

Synchrotron SAXS measurements were collected at beamline 18-ID (BioCAT) of the Advanced Photon Source with in-line size exclusion chromatography (SEC-SAXS) to separate sample from aggregates and other contaminants thus ensuring optimal sample quality. For each SEC-SAXS run, 300 µL of 8 mg mL^{-1} SenB in 25 mM Tris, 100 mM NaCl, 10% glycerol, pH 8 was loaded onto a Superdex 200 Increase 10/300 GL column (Cytiva), which was run at 0.4 mL min^{-1} by an AKTA Pure FPLC (GE) and the eluate flown through the SAXS flow cell after it passed through the UV monitor. The flow cell consisted of a 1.0 mm ID quartz capillary with ~20 µm walls. A coflowing buffer sheath was used to separate sample from the capillary walls, helping prevent radiation damage.[17] For the sample containing 10 eq. of UDP-GlcNAc, 110 µM UDP-GlcNAc was included in the SEC buffer to ensure ligand binding throughout the experiment. To ensure protein stability, the cell and column were both at 5 °C for the duration of all experiments. Data were collected with an incident wavelength of 1.033 Å and scattering intensities were recorded using a Pilatus3 X 1M detector which was placed 3.682 m from the sample, giving access to a q-range of 0.0027 $Å^{-1}$ to 0.33 $Å^{-1}$. 0.5 s exposures were acquired every 1 s during elution and data was reduced using BioXTAS RAW 2.1.4.[18] Radial averaging, frame comparison, averaging, subtraction, Guinier fit, and M.W. analysis were also done using BioXTAS RAW 2.1.4.[19-21] Buffer blanks were created by averaging regions flanking the elution peak and subtracted from exposures selected from the elution peak to create the I(q) vs. q curves used for subsequent analyses. Within BioXTAS RAW 2.1.4, D_{max} and $P(r)$ analyses were calculated from the experimental scattering curves using GNOM[22] and *ab initio* electron density maps that were refined to produce the final scattering envelopes were created using DENSS (DENsity from Solution Scattering).[23] The DENSS Alignment tool in BioXTAS RAW was used to align the SenB crystal structure with the substrate-free electron density reconstruction (Fig. S5C). Note that for the UDP-GlcNAc-bound reconstruction, the simulated closed conformation of SenB was used for alignment. Selected data processing statistics can be found in Table S3.

***In Silico* Docking**

The UDP- and SeP-bound docking model of SenB was generated using the High Ambiguity Driven protein-protein DOCKing (HADDOCK) 2.4 server.[24] Our crystallographic model has several residues within the active site cleft that were not resolved due to a lack of electron density, suggesting that there is conformational flexibility in the absence of substrate. To perform molecular docking, we first built in missing residues using Coot and replaced stubbed residues with full-length side chains. Gaps in the crystallographic model are 1-6 residues in length. While electron density is poor in these regions, it was sufficient for building the backbone with modest confidence. Note that regions with missing density were specified as fully flexible in the docking simulations. Another challenging aspect regarding ligand docking to the substrate-free SenB structure is its putative catalytically inactive open conformation. To simulate UDP/UDP-GlcNAc-induced domain and/or loop movement to generate the catalytically active state, three-body docking was performed with UDP, after separating the NTD (residues 1-130) and the CTD (residues 131-331) of the SenB crystal structure into two PDB files. Center of mass restraints (with a force constant of 1.0) were turned on to enforce contact between the separated domains, and connectivity restraints were supplied to maintain connectivity by specifying an unambiguous distance restraint of 2.0 Å between residues 130 and 131.[25] In addition to specifying residues lacking electron density as fully flexible, the half of the C-terminal α-helix that binds to the NTD was also specified as a fully flexible region.

Following generation of a closed structure of SenB, residues 130 and 131 were reconnected in Coot, and two-body docking was repeated sequentially with UDP then SeP. The simulations were run with the default parameters for protein-ligand interactions, excluding the random removal of AIRs. Comparison of the closed SenB conformation with UDP- and phosphate-bound structures of structurally homologous glycosyltransferases (see Table S2) were used to aid in prediction of binding residues and define the ambiguous interaction restraints (AIRs) for the docking simulations.

Supplementary Tables

Table S1. Crystallographic data processing and refinement statistics for SenB.

**PDB ID**	8FBX
**Data Collection**[a]	
Space group	P 1 2 1
Unit cell (Å)	a = 93.00, b = 68.56, c = 93.57<br>α = γ = 90.00, β = 118.13
Wavelength (Å)	0.9785
Resolution range (Å)	40.01 – 2.25 (2.33 – 2.25)
Total observations	168319 (17103)
Total unique observations	48509 (4189)
$CC_{1/2}$	0.985 (0.779)
I/σ_1	8.31 (2.52)
Completeness (%)	93.16 (84.91)
R_{merge}	0.1205 (0.6042)
R_{pim}	0.07633 (0.3696)
R_{meas}	0.1432 (0.71)
Redundancy	3.5 (3.6)
Wilson B-factor	28.57
**Refinement Statistics**	
Reflections (test)	1730 (150)
Total atoms refined	7076
Solvent	418
R_{work} (R_{free})	0.2020 (0.2383)
RMSDs	
Bond lengths (Å)/angles (°)	0.013/1.45
Ramachandran plot	
Favored/allowed (%)	97.94/2.06
Mean B values (Å^2)	
Protein	31.00
Ligands	45.98
Solvent	33.72

[a] Values in parentheses refer to the high-resolution shell.

Table S2. Top ranking structures (ranked by the Dali Z-score) homologous to SenB, as identified by the Dali protein structure comparison server.

PDB ID	Chain	Dali Z-score	RMSD Cα [Å]	% Identity	Description (donor organism)	Ref
2JJM	D	27.5	3.0	12	Family GT4 glycosyltransferase (*Bacillus anthracis*)	(26)
3MBO	A	27.4	3.1	12	Glycosyltransferase BshA (*Bacillus anthracis*)	(27)
6D9T	A	27.4	3.0	11	Glycosyltransferase BshA (*Staphylococcus aureus*)	(28)
6KIH	L	27.2	2.6	18	Sucrose phosphate synthase (*Thermosynechococcus elongatus*)	(29)
6TVP	A	26.8	3.4	16	Alpha-maltose-1-phosphate synthase GlgM (*Mycolicibacterium smegmatis*)	(30)
5D01	B	26.6	3.0	16	Glycosyltransferase BshA (*Bacillus subtilis*)	(31)
2GEJ	A	26.6	3.0	15	Mannosyltransferase PimA (*Mycolicibacterium smegmatis*)	(32)
3C4V	A	26.2	3.0	17	Glycosyltransferase MshA (*Corynebacterium glutamicum*)	(33)
3FRO	B	26.1	3.0	14	Glycogen synthase (*Pyrococcus abyssi*)	(34)
4XSP	A	26.1	2.7	16	Glucosyltransferase Alr3699/HepE (*Anabaena* sp. strain PCC 7120)	(35)
6EJJ	B	25.6	2.9	15	Glycosyltransferase PglH (*Campylobacter jejuni*)	(36)
3OKC	A	25.3	3.4	14	Mannosyltransferase PimB (*Corynebacterium glutamicum*)	(37)

Table S3. SAXS data processing results.

	Apo-SenB	UDP-GlcNAc-bound SenB
**Guinier Analysis**		
I(0) (cm^{-1})	0.018 +/- 4.93e-5	0.023 +/- 3.87e-5
R_g (Å)	23.91 +/- 0.12	22.84 +/- 0.067
$q_{min}R_g$ (Å^{-1})	0.078	0.12
$q_{max}R_g$ (Å^{-1})	1.30	1.34
Coefficient of correlation, R^2	0.97	0.99
**P(r) Analysis**		
I(0) (cm^{-1})	0.018 +/- 5.59e-5	0.023 +/- 4.42e-5
R_g (Å)	24.44 +/- 0.17	23.36 +/- 0.095
D_{max} (Å)	95.0	85.0
q range (Å^{-1})	0.0033 to 0.3327	0.0052 to 0.3327
χ^2 (total estimate from GNOM)	0.99 (0.76)	1.03 (0.83)
Porod volume (nm^3)	41.9	38.9
Molecular weight (kDa), calculated from the Porod volume	34.7	32.3

Supplementary Figures

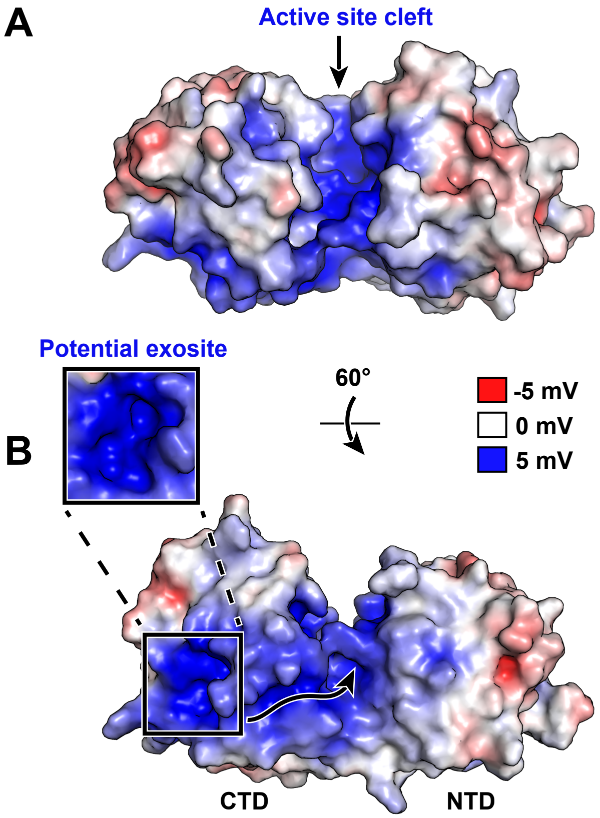

Figure S1. Electrostatic surface representation of SenB. (A) The interdomain cleft of SenB is highly electropositive, suitable for binding to the negatively charged substrates. (B) A second positively charged pocket, located ~20 Å away, is connected to the active site by an electropositive groove.

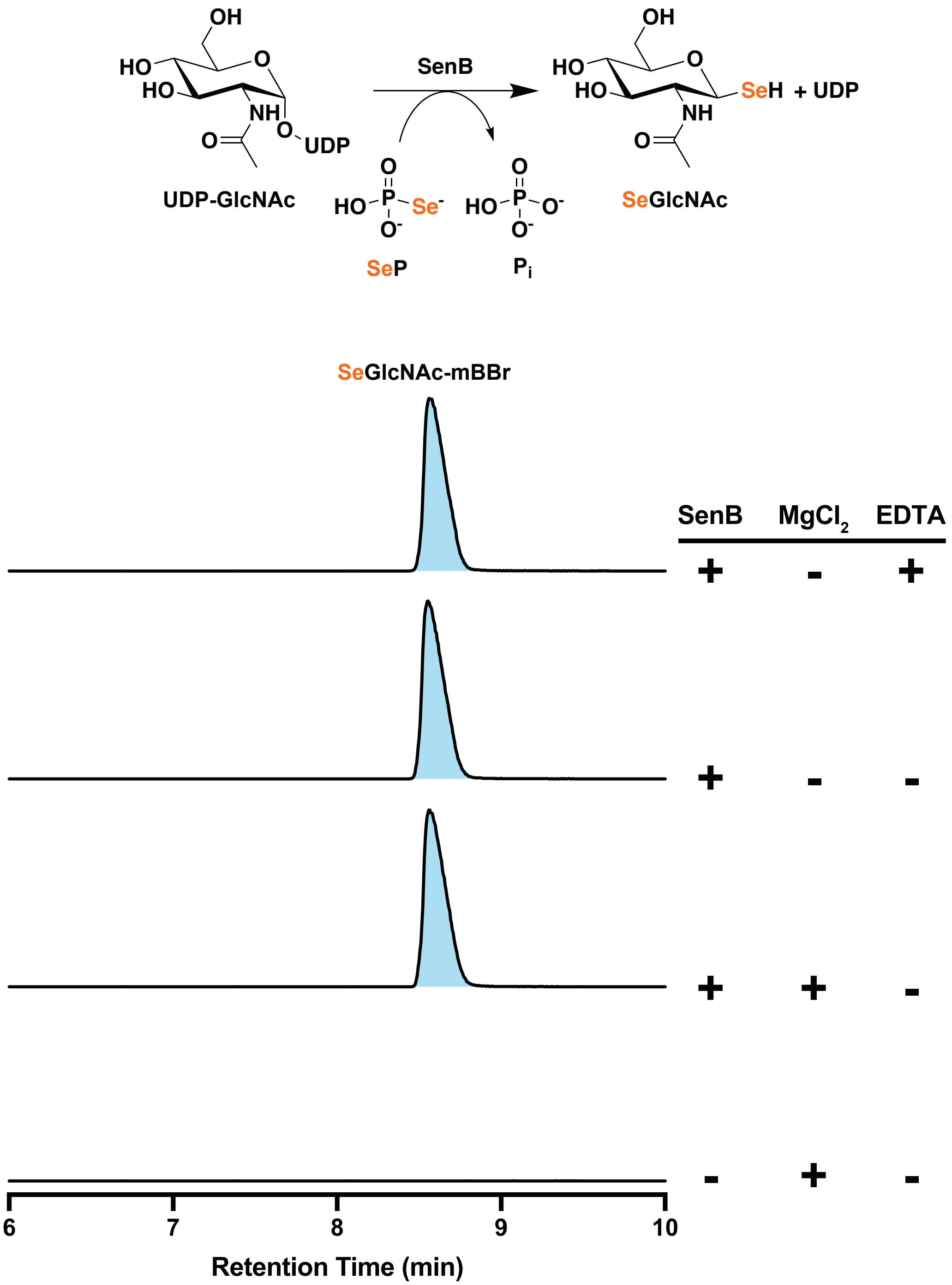

Figure S2. SenB metal-dependence assays. Reactions were carried out in 50 mM Tris buffer (pH 8) containing 2 mM DTT, 1 mM UDP-GlcNAc, 1 mM synthetic SeP, 0 or 1 mM $MgCl_2$, 0 or 1 mM EDTA, and 0 or 20 µM SenB. After 10 min, reactions were derivatized with mBBr and analyzed by HPLC-MS. Extracted ion chromatograms for mBBr-derivatized SeGlcNAc are shown.

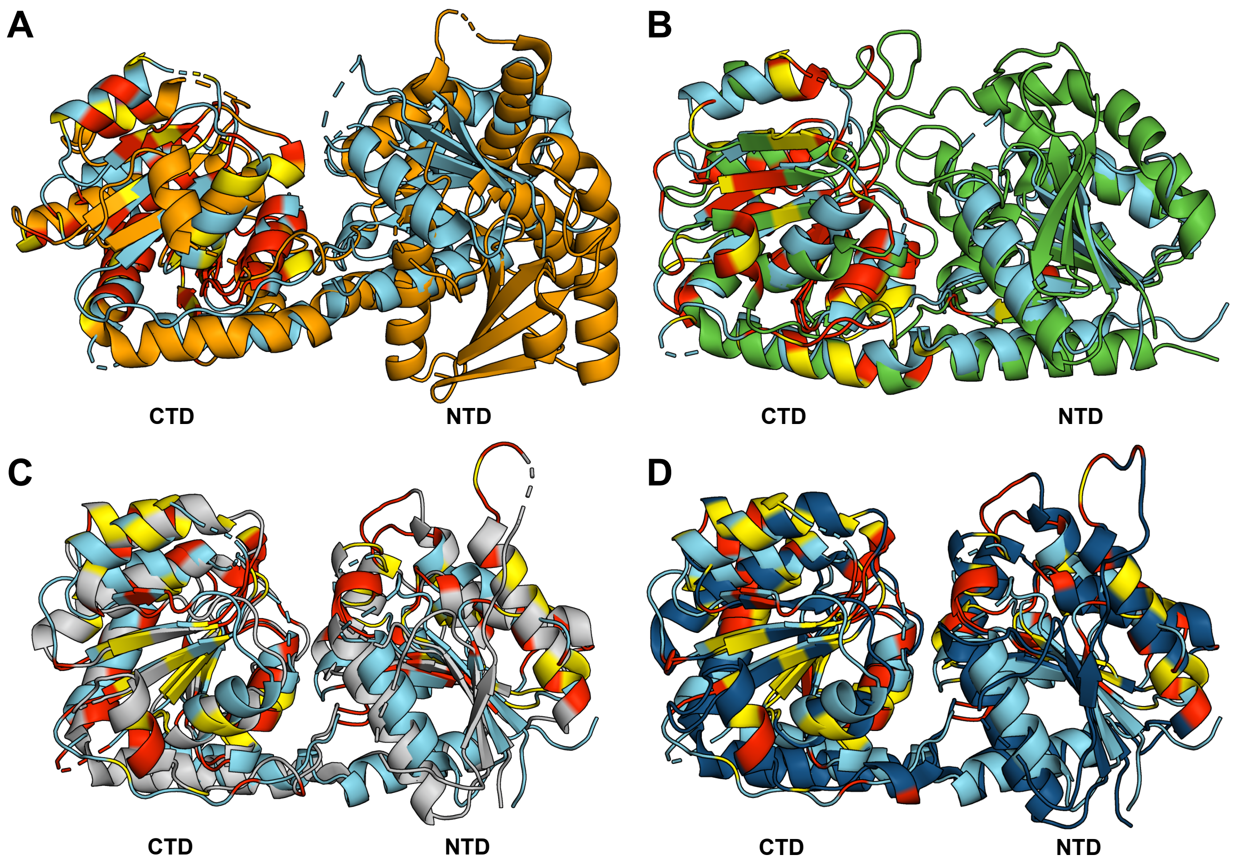

Figure S3. Comparison of SenB with structural homologues. Alignment of SenB (light blue) and (A) MshA (orange, PDB ID: 3C48), (B) *T. elongatus* sucrose phosphate synthase bound to UDP (green, PDB ID: 6KIH), (C) *B. anthracis* family GT4 glycosyltransferase (gray, PDB ID: 2JJM), and (D) *B. anthracis* glycosyltransferase BshA bound to UDP (dark blue, PDB ID: 3MBO). Conserved residues are shown in red and yellow (identical residues shown in red, residues with similar biochemical characteristics shown in yellow). Note that only in MshA are the conserved residues with SenB fully localized to the nucleotide-sugar-binding C-terminal domain (left). The stark difference between the two domains can be ascribed to both enzymes utilizing UDP-GlcNAc as the preferred donor substrate, which binds predominantly to residues in the CTD and the highly divergent acceptor substrates (SeP for SenB, 1-L-myo-inositol 1-phosphate for MshA), which bind predominantly to NTD residues.

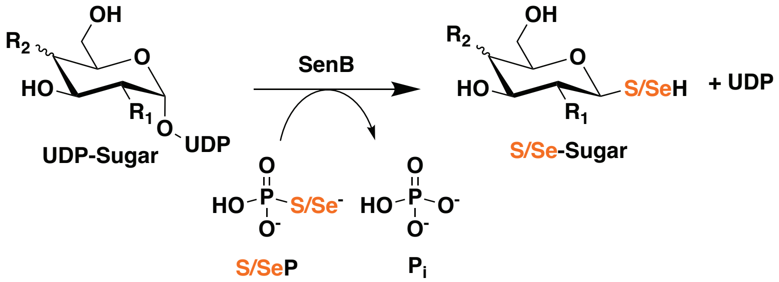

	R_1	R_2
UDP-Glc	-OH	-$OH_{equatorial}$
UDP-GlcNAc	-NHAc	-$OH_{equatorial}$
UDP-GalNAc	-NHAc	-OH_{axial}

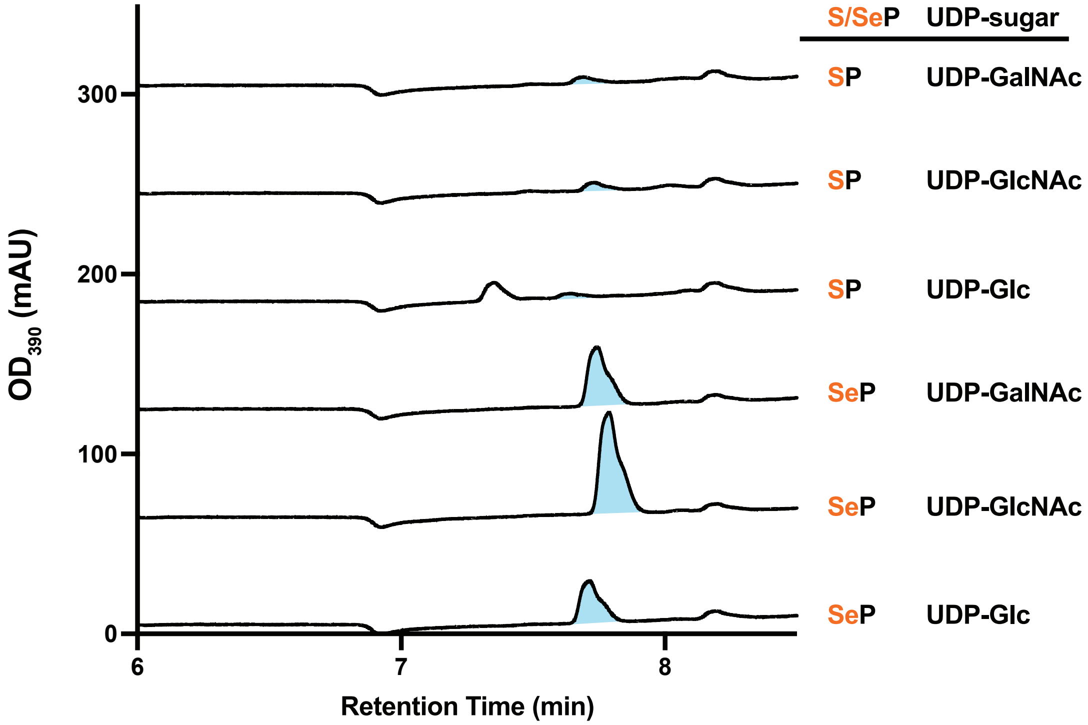

Figure S4. SenB selenophosphate/thiophosphate preference assays. Reactions were carried out in 50 mM tricine buffer (pH 7.2) containing 20 mM KCl, 5 mM $MgCl_2$, 2 mM DTT, 2 mM UDP-sugar, and 20 µM SenB. Reactions included either 1 mM sodium thiophosphate (SP) or 2 mM ATP, 1 mM Na_2Se, and 20 µM SenC to generate selenophosphate (SeP). After 2 h, reactions were derivatized with mBBr and analyzed by HPLC, monitoring absorbance at 390 nm. Blue-filled peaks correspond to derivatized selenosugar/thiosugar enzyme products. Integrated peak areas of derivatized selenosugars are >10 times greater than that of their corresponding thiosugars, demonstrating SenB's preference for selenophosphate over thiophosphate.

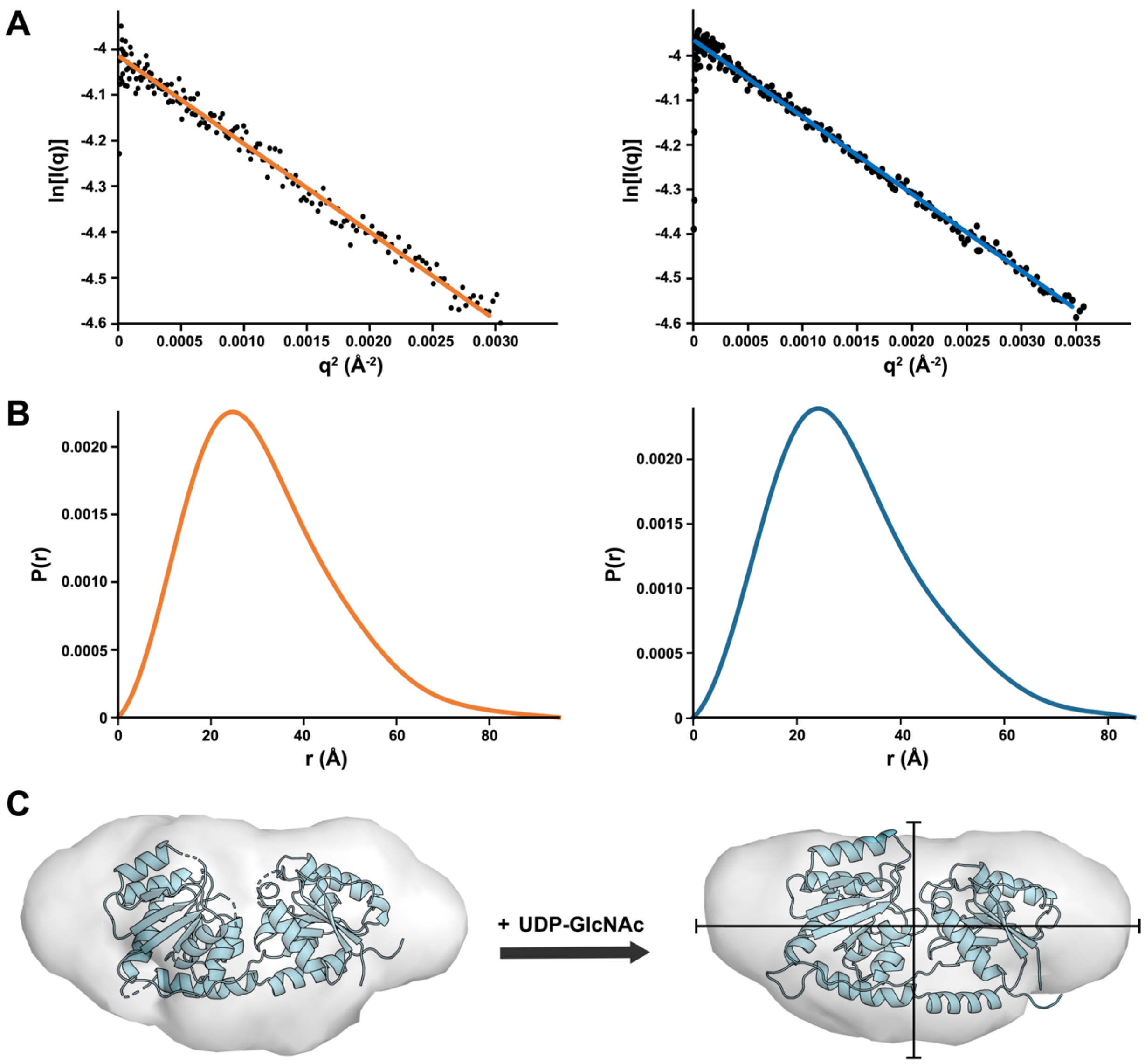

Figure S5. SAXS analysis for substrate-free and UDP-GlcNAc-bound SenB. (A) Guinier plots of apo-SenB (left) and SenB bound to UDP-GlcNAc (right). Both samples indicate no aggregation or repulsive interparticle interactions, as evidenced by a flat linear regression. (B) *P*(*r*) functions of apo-SenB (left) and SenB bound to UDP-GlcNAc (right). (C) SAXS 3D electron density reconstructions for apo-SenB (left) and UDP-GlcNAc-bound SenB (right). The crystal structure of apo-SenB is aligned to the substrate-free envelope and the simulated closed SenB structure aligned to the UDP-GlcNAc-bound envelope. The black lines over the substrate-bound-SenB envelope represent the dimensions of the apo-SenB envelope, demonstrating a slight reduction in size with ligand binding.

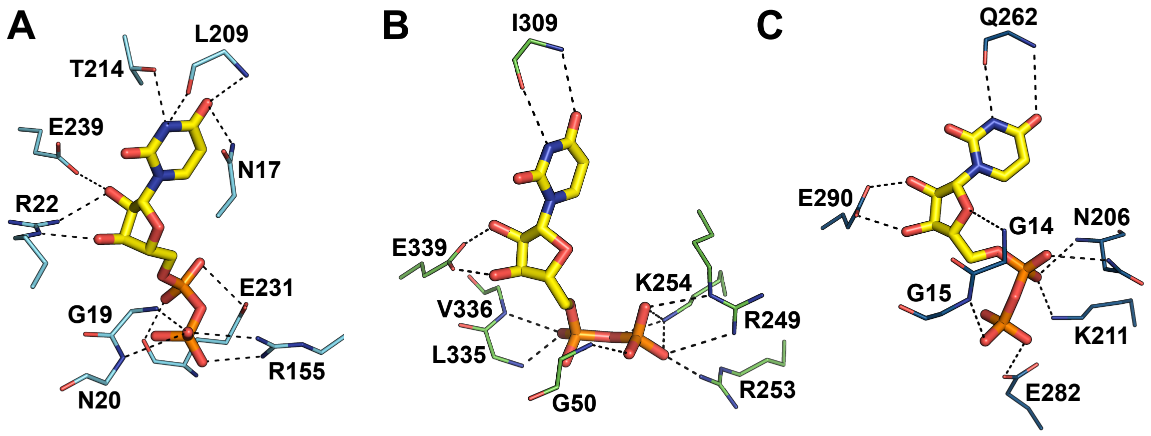

Figure S6. Structural comparison of UDP-binding contacts. Shown are (A) the SenB docking model, along with structures of (B) SPS/UDP (PDB ID: 6KIH) and (C) BshA/UDP (PDB ID: 3MBO).

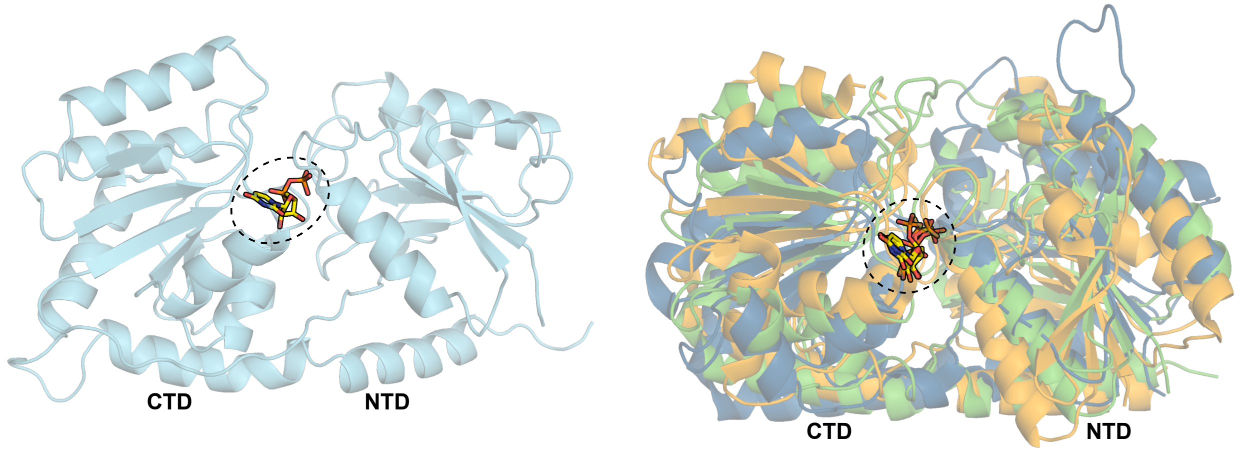

Figure S7. UDP binding in SenB and structural homologues. The SenB docking model (left, light blue) has UDP positioned in a similar orientation within the interdomain cleft compared to MshA (orange, PDB ID: 3C4V), SPS (green, PDB ID: 6KIH), and BshA (dark blue, PDB ID: 3MBO).

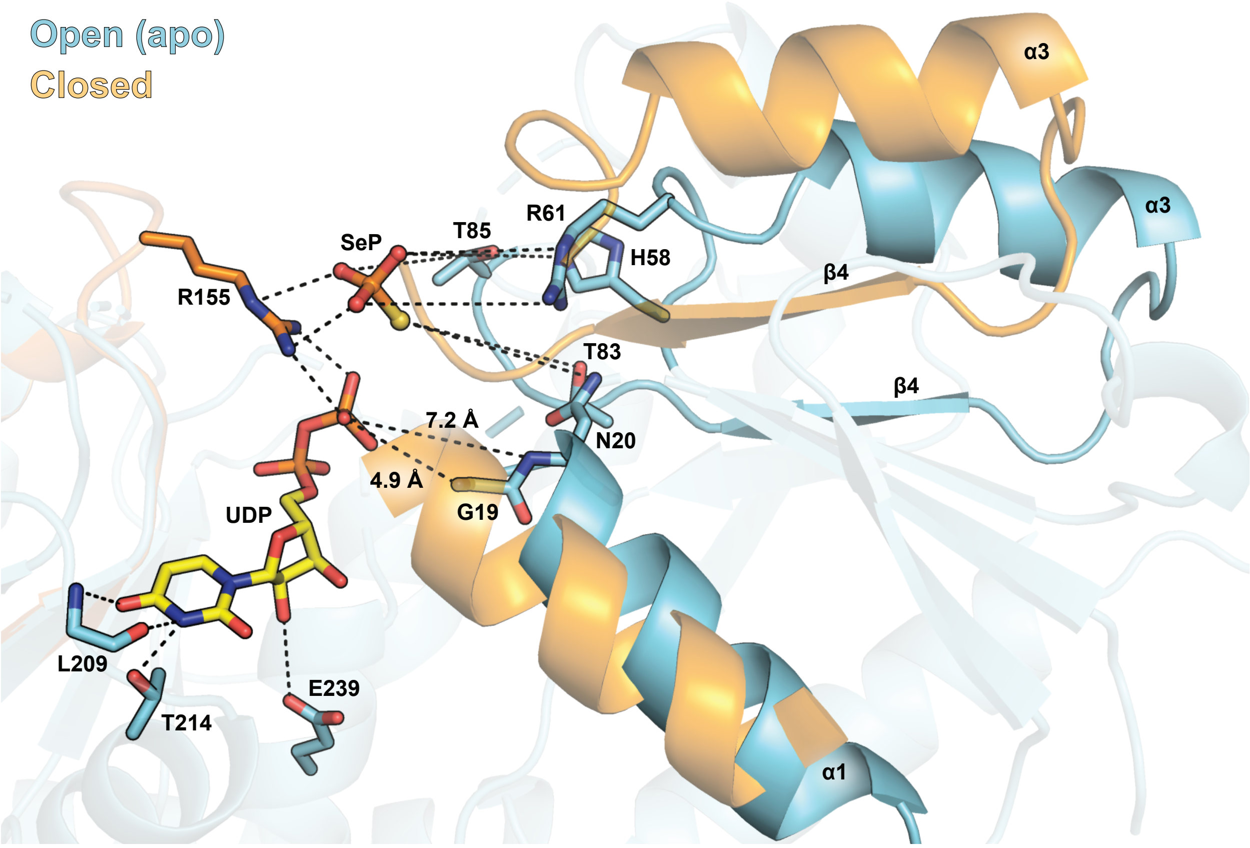

Figure S8. SenB's NTD UDP- and SeP-binding contacts are possible only in the closed conformation. The substrate-free SenB crystal structure is depicted in blue, and the UDP + SeP docking model shown in orange. Alignment of the CTDs reveals that in the open conformation, the H-bonds between UDP and NTD residues G19 and N20 are no longer energetically favorable (4.9 and 7.2 Å, respectively) due to rotation of the first α-helix (α1). The CTD UDP-binding contacts (L209, T214, and E239), however, remain in the open conformation. Rotation of α3 and β4 in the NTD also causes the H-bonds between docked SeP and NTD residues N20, H58, R61, T83, and T85 to become energetically unfavorable, increasing their distances to 7.9, 9.3, 6.5/8.6, 9.0, and 8.7 Å, respectively. The side chain of R155 in the closed conformation (orange) is shown, demonstrating that in order for SeP to bind to R155 and its NTD contacts, domain rotation as predicted by our model is necessary. Residues N17, R22, R155, and E231 are not shown in the open conformation due to missing density in the substrate-free structure that prohibited modeling of their side chains.

Supplementary References

(1) Kayrouz, C. M., Huang, J., Hauser, N., and Seyedsayamdost, M. R. (2022) Biosynthesis of selenium-containing small molecules in diverse microorganisms, *Nature 610*, 199−204.

(2) Borecka, B., Chojnowski, J., Cypryk, M., Michalski, J., and Zielinska, J. (1979) Synthetic and mechanistic aspects of the reaction of trialkylsilyl halides with thio and seleno esters of phosphorus, *J. Organomet. Chem. 171*, 17−34.

(3) Kamiñski, R., Glass, R. S., and Skowroñska, A. (2001) A convenient synthesis of selenocarboxamides from nitriles, *Synthesis 33*, 1308−1310.

(4) Johnson, M., Zaretskaya, I., Raytselis, Y., Merezhuk, Y., McGinnis, S., and Madden, T. L. (2008) NCBI BLAST: a better web interface, *Nucleic Acids Res. 36*, 5−9.

(5) Breton, C., Snajdrova, L., Jeanneau, C., Koca, J., and Imberty, A. (2006) Structures and mechanisms of glycosyltransferases, *Glycobiology 16*, 29−37.

(6) Qasba, P. K., Ramakrishnan, B., and Boeggeman, E. (2005) Substrate-induced conformational changes in glycosyltransferases, *Trends Biochem. Sci. 30*, 53−62.

(7) Kabsch, W. (2010) XDS, *Acta Crystallogr. D Biol. Crystallogr. 66*, 125−132.

(8) Evans, P. R., and Murshudov, G. N. (2013) How good are my data and what is the resolution?, *Acta Crystallogr. D Biol. Crystallogr. 69*, 1204−1214.

(9) Jumper, J., Evans, R., Pritzel, A., Green, T., Figurnov, M., Ronneberger, O., et al. (2021) Highly accurate protein structure prediction with AlphaFold, *Nature 596*, 583−589.

(10) McCoy, A. J., Sammito, M. D., and Read, R. J. (2022) Implications of AlphaFold2 for crystallographic phasing by molecular replacement, *Acta Crystallogr. D Struct. Biol. 78*, 1−13.

(11) Barbarin-Bocahu, I., and Graille, M. (2022) The X-ray crystallography phase problem solved thanks to AlphaFold and RoseTTAFold models: a case-study report, *Acta Crystallogr. D Struct. Biol. 78*, 517−531.

(12) Emsley, P., Lohkamp, B., Scott, W. G., and Cowtan, K. (2010) Features and development of Coot, *Acta Crystallogr. D Biol. Crystallogr. 66*, 486−501.

(13) Adams, P. D., Afonine, P. V., Bunkoczi, G., Chen, V. B., Davis, I. W., Echols, N., et al. (2010) PHENIX: a comprehensive python-based system for macromolecular structure solution, *Acta Crystallogr. D Biol. Crystallogr. 66*, 213−221.

(14) Williams, C. J., Headd, J. J., Moriarty, N. W., Prisant, M. G., Videau, L. L., Deis, L. N., et al. (2018) MolProbity: more and better reference data for improved all-atom structure validation, *Protein Sci. 27*, 293−315.

(15) Schrodinger, LLC. (2023) The PyMOL molecular graphics system, version 2.5.5.

(16) Jurrus, E., Engel, D., Star, K., Monson, K., Brandi, J., Felberg, L. E., et al. (2018) Improvements to the APBS biomolecular solvation software suite, *Protein Sci. 27*, 112−128.

(17) Kirby, N., Cowieson, N., Hawley, A. M., Mudie, S. T., McGillivray, D. J., Kusel, M., et al. (2016) Improved radiation dose efficiency in solution SAXS using a sheath flow sample environment, *Acta Crystallogr. D Struct. Biol. 72*, 1254−1266.

(18) Hopkins, J. B., Gillilan, R. E., and Skou, S. (2017) BioXTAS RAW: improvements to a free open-source program for small-angle X-ray scattering data reduction and analysis, *J. Appl. Crystallogr. 50*, 1545−1553.

(19) Rambo, R. P., and Tainer, J. A. (2013) Accurate assessment of mass, models and resolution by small-angle scattering, *Nature 496*, 477−481.

(20) Piiadov, V., Ares de Araujo, E., Oliveira Neto, M., Craievich, A. F., and Polikarpov, I. (2019) SAXSMoW 2.0: online calculator of the molecular weight of proteins in dilute solution from experimental SAXS data measured on a relative scale, *Protein Sci. 28*, 454−463.

(21) Fischer, H., de Oliveira Neto, M., Napolitano, H. B., Polikarpov, I., and Craievich, A. F. (2009) Determination of the molecular weight of proteins in solution from a single small-angle X-ray scattering measurement on a relative scale, *J. Appl. Crystallogr. 43*, 101−109.
(22) Svergun, D. I. (1992) Determination of the regularization parameter in indirect-transform methods using perceptual criteria, *J. Appl. Cryst. 25*, 495−503.
(23) Grant, T. D. (2018) Ab initio electron density determination directly from solution scattering data, *Nat. Methods 15*, 191−193.
(24) Dominguez, C., Boelens, R., and Bonvin, A. M. J. J. (2003) HADDOCK: a protein-protein docking approach based on biochemical or biophysical information, *J. Am. Chem. Soc. 125*, 1731−1737.
(25) Karaca, E., and Bonvin, A. M. (2011) A multidomain flexible docking approach to deal with large conformational changes in the modeling of biomolecular complexes, *Structure 19*, 555−565.
(26) Ruane, K. M., Davies, G. J., and Martinez-Fleites, C. (2008) Crystal structure of a family GT4 glycosyltransferase from Bacillus anthracis ORF BA1558, *Proteins 73*, 784−787.
(27) Parsonage, D., Newton, G. L., Holder, R. C., Wallace, B. D., Paige, C., Hamilton, C. J., et al. (2010) Characterization of the N-acetyl-alpha-D-glucosaminyl l-malate synthase and deacetylase functions for bacillithiol biosynthesis in Bacillus anthracis, *Biochemistry 49*, 8398−8414.
(28) Royer, C. J., and Cook, P. D. (2019) A structural and functional analysis of the glycosyltransferase BshA from Staphylococcus aureus: insights into the reaction mechanism and regulation of bacillithiol production, *Protein Sci. 28*, 1083−1094.
(29) Li, Y., Yao, Y., Yang, G., Tang, J., Ayala, G. J., Li, X., et al. (2020) Co-crystal structure of Thermosynechococcus elongatus sucrose phosphate synthase with UDP and sucrose-6-phosphate provides insight into its mechanism of action involving an oxocarbenium ion and the glycosidic bond, *Front. Microbiol. 11*, 1050−1064.
(30) Syson, K., Stevenson, C. E. M., Lawson, D. M., and Bornemann, S. (2020) Structure of the Mycobacterium smegmatis alpha-maltose-1-phosphate synthase GlgM, *Acta Crystallogr. F Struct. Biol. Commun. 76*, 175−181.
(31) Winchell, K. R., Egeler, P. W., VanDuinen, A. J., Jackson, L. B., Karpen, M. E., and Cook, P. D. (2016) A structural, functional, and computational analysis of BshA, the first enzyme in the bacillithiol biosynthesis pathway, *Biochemistry 55*, 4654−4665.
(32) Guerin, M. E., Kordulakova, J., Schaeffer, F., Svetlikova, Z., Buschiazzo, A., Giganti, D., et al. (2007) Molecular recognition and interfacial catalysis by the essential phosphatidylinositol mannosyltransferase PimA from mycobacteria, *J. Biol. Chem. 282*, 20705−20714.
(33) Vetting, M. W., Frantom, P. A., and Blanchard, J. S. (2008) Structural and enzymatic analysis of MshA from Corynebacterium glutamicum: substrate-assisted catalysis, *J. Biol. Chem. 283*, 15834−15844.
(34) Diaz, A., Diaz-Lobo, M., Grados, E., Guinovart, J. J., Fita, I., and Ferrer, J. C. (2012) Lyase activity of glycogen synthase: is an elimination/addition mechanism a possible reaction pathway for retaining glycosyltransferases?, *IUBMB Life 64*, 649−658.
(35) Wang, X. P., Jiang, Y. L., Dai, Y. N., Cheng, W., Chen, Y., and Zhou, C. Z. (2016) Structural and enzymatic analyses of a glucosyltransferase Alr3699/HepE involved in Anabaena heterocyst envelop polysaccharide biosynthesis, *Glycobiology 26*, 520−531.
(36) Ramirez, A. S., Boilevin, J., Mehdipour, A. R., Hummer, G., Darbre, T., Reymond, J. L., and Locher, K. P. (2018) Structural basis of the molecular ruler mechanism of a bacterial glycosyltransferase, *Nat. Commun. 9*, 445−455.
(37) Batt, S. M., Jabeen, T., Mishra, A. K., Veerapen, N., Krumbach, K., Eggeling, L., et al. (2010) Acceptor substrate discrimination in phosphatidyl-myo-inositol mannoside synthesis: structural and mutational analysis of mannosyltransferase Corynebacterium glutamicum PimB, *J. Biol. Chem. 285*, 37741−37752.
